# Socio-economic determinants of disease progression among HIV infected adults in Kenya

**DOI:** 10.1186/s12889-015-2084-8

**Published:** 2015-07-31

**Authors:** Nyawira T. Gitahi–Kamau, James N. Kiarie, Kenneth K. Mutai, Beatrice W. Gatumia, P. M. Gatongi, A. Lakati

**Affiliations:** University of Nairobi, College of Health sciences, Kenyatta National Hospital, P.O. Box 19676 – 00202, Nairobi, Kenya; Moi University, School of Medicine, P.O Box 4606, Eldoret, Kenya; Directorate of Capacity Building -AMREF International Training Programme, Nairobi, Kenya; Kenyatta National Hospital, Respiratory and infectious disease unit, Hospital Road, Upper Hill, Nairobi, Kenya; Kenya Heart Foundation, Nairobi, Kenya

**Keywords:** Discordant, Highly active antiretroviral therapy, Income, Socio-economic determinants, Disease progression

## Abstract

**Background:**

Socioeconomic determinants have been shown to have an effect on the progression of HIV disease evidenced by studies carried out largely in developed countries. Knowledge of these factors could inform on prioritization of populations during scale up of highly active antiretroviral therapy (HAART) constrained health systems. The objective of this study was to identify socioeconomic correlates of HIV disease progression in an adult Kenyan population.

**Methods:**

We analysed data from 312 HIV positive individuals, drawn from a cohort enrolled in a randomized clinical trial investigating the effectiveness of Acyclovir in the prevention of HIV transmission among serodiscordant couples. In this study we included individuals with CD4 counts ≥ 350 cells/mm^3^ and World Health Organization (WHO), clinical stage one or two. The exposure variables measured were: - daily household income available for expenditure, age, gender, housing type and level of formal education. We used a composite outcome of disease progression to WHO clinical stage 3 or 4 or a laboratory outcome of CD4 count below 350 cells/mm^3^ after two years of follow-up. Logistic regression was used to determine associations of variables that were found to be significant at univariate analysis, and to control for potential confounders.

**Results:**

Seventy eight (25 %) individuals reported HIV disease progression. Majority (79.9 %) were female. The median age was 30 year and 93.6 % had attained a primary level of education. Median CD4 at enrolment into the clinical trial was 564 cells/mm^3^; those who had disease progression were enrolled with a significantly (*p* < 0.001) lower CD4 count. Daily household income available for expenditure adjusted for CD4 count at enrolment was associated significantly (*p* = 0.04) with HIV disease progression. Disease progression was five times more likely to occur in study subjects with daily income available for expenditure of less than US$1 compared to those with more than US$ 5 available for daily expenditure [adjusted Odds Ratio 4.6 (95 % Confidence Interval 1.4–14.4)]. Disease progression was not associated with age, gender, type of housing or level of education attained (*p* < 0.05).

**Conclusion:**

Populations with low household incomes should be considered vulnerable to disease progression and should therefore be prioritized during the scale up of HAART for treatment as prevention.

## Background

HIV disease progression to Acquired Immune Deficiency Syndrome (AIDS) is one of the greatest contributors of mortality in Africa [1]. A large number of infections occur among serodiscordant couples [2]. The progressive loss of CD4+ T lymphocytes during HIV infection eventually results in an inability to mount an adequate immune response to opportunistic pathogens resulting in death [3].

Socioeconomic determinants such as age, sex, and income are reported as contributors to disease progression [4–8] in HIV infected individuals who are not on highly active antiretroviral therapy (HAART). These factors may still continue to influence the uptake of care and treatment even, where these services are provided at no cost to the individual. There is a paucity of data on the effects of socioeconomic determinants of disease progression among HIV infected individuals in sub-Saharan Africa.

The World Health Organization (WHO) recommends the treatment of all HIV positive individuals in a discordant relationship regardless of their CD4 count and all adults and adolescents with CD4 cell counts below 500 cells/mm^3^ [9] should receive treatment. The implementation of these guidelines [9] in low-income countries like Kenya [10] may be hindered due to the financial considerations on already strained health systems. To alleviate this, low and middle-income countries commonly use a phasic approach in the implementation of HAART scaling up [11]. Integration of clinical and sociodemographic criteria would be useful in the in prioritization of vulnerable populations. The objective of this study was to identify socioeconomic correlates of disease progression in HIV infected adults in serodiscordant relationships.

## Methods

**Fig. 1:**
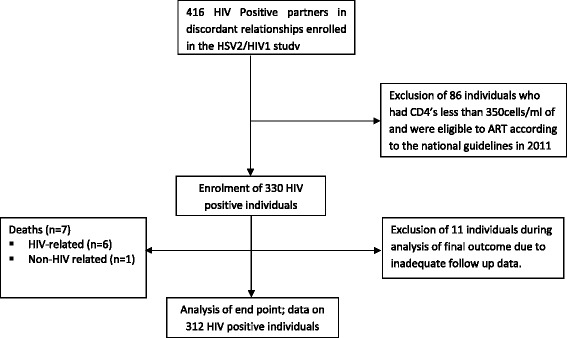
Cohort selection

This study was conducted in a sample of HIV positive individuals drawn from a randomized clinical trial (RCT) carried out in a serodiscordant couple’s clinic in Nairobi. The RCT was investigating Acyclovir in the prevention of HIV transmission (HSV2/HIV1 study) - clinical trials no: NCT00194519 [12]. We analysed data from 312 HIV positive individuals drawn from a cohort of 416 study participants for a period of two years beginning in November 2011 and ending in June 2013. Participants were selected from the main cohort based on CD4 count of above 350 cells/mm^3^ and WHO clinical stage 1 or 2 at enrolment into the clinical trial. We excluded HIV positive individuals who seroconverted during the clinical trial [10]. Out of the total cohort initially enrolled in the clinical trial, 86 were excluded due to CD4 counts of ≤350 cells/mm^3^ at enrollment, because they were eligible for HAART according to the Kenya national guidelines 2011 [13]. End point outcomes for 18 participants (5 %) were unavailable because they were reported as lost to follow up (*n* = 11) or dead (*n* = 7) during the clinical trial. A summary of the cohort is illustrated in Fig. 1.

Disease progression was defined as a composite outcome of WHO clinical staging criteria stages 3 and 4 or CD4 count of < 350 cells/mm^3.^ Outcomes were evaluated at six month intervals over the two years. Socio-demographic and economic predictors of disease investigated included daily income available for expenditure, level of education, housing settlement, age, and sex.

### Data analysis

Statistical analysis was performed using SPSS version 17.0. The main outcome was HIV disease progression which was a composite outcome of CD4 less than 350 cells/mm^3^ and/or WHO clinical stage 3 or 4 within the 2-year follow up period. Available daily income for expenditure was categorized as < US$1, US$1–5 and > US$5 .We took into account taking the World Bank (WB) definitions of extreme poverty and poverty as having daily income available for expenditure below US$1.25 and below $2 respectively. We also allowed for the variations that occur within countries [14]. Chi-square tests were used to determine differences in disease progression (by both WHO staging and CD4 count criteria) across demographic, socioeconomic, and clinical characteristics. Mann Whitney *U* test was used to compare median CD4 at enrolment between the two disease progression groups. Logistic regression was carried out to determine independent predictors of HIV disease progression. CD4 count at enrolment (baseline) was a known confounder of disease progression and was included in the regression model after normalization through cube root transformation. All statistical tests were significant at a *p* value ≤0.05.

### Ethical considerations

The HSV2/HIV1 study involved rigorous informed consent and counselling sessions during the enrolment of participants. Ethical approval was obtained from the Kenyatta National Hospital ethics research committee and the institutional regulatory board of University of Washington. The approvals allowed for the use of archived data and stored samples for future studies. The current study was approved by the Moi University Institutional regulatory and ethics committee.

## Results

### Population characteristics

The 312 individuals analyzed, entered the study in early HIV disease; characterized as WHO stage 1 or 2, or a CD4 count ≥350 cells/mm^3^. Eighty percent of all the study participants were female. Median age was 30 years (Interquartile Range [IQR]; 26, 35) with majority (91 %), below 40 years. The median household daily income for expenditure was $3 (IQR 1.25, 7). The population was highly literate with only 6.4 % reporting no basic education. Majority (61.2 %) of the participants lived in formal housing settlements. A summary of the background characteristics is shown in Table 1.

**Table 1:** Baseline characteristics of study population *n* = 312

Variable	Number	Percent
Sex		
Males	61	20.3
Females	251	79.9
Age in years		
15-24	54	17.3
25-34	175	56
35-44	65	20.8
45-54	12	3.9
55-64	3	0.1
65-74	3	0.1
Level of education		
Primary	128	41
Sec school	111	35.6
Tertiary	53	17
No education	20	6.4
Daily household income available for expenditure		
<1$	135	43.3
1-5$	131	41.9
>5$	46	14.8
Housing settlement		
Informal	116	37.2
Formal	191	61.2
Not sure	5	1.6
Treatment arm		
Placebo	142	43
Acyclovir	170	51.5
Baseline CD4 count		
350-500	121	38.8
501-750	106	34.0
750	85	27.2
WHO stage 1 and 2	312	

### Disease progression

HIV disease progression occurred in 25 % (95 % CI 20.5–30.1 %) of the participants; 20.5 % showed progression based on CD4 count criteria (<350 cells/mm^3^) and 4.5 % depicted progression by WHO clinical staging criteria alone. During the two year follow up there were four reported deaths; three of which were from HIV associated conditions and one with no definitive diagnosis. Median CD4 count at baseline was 566 cells/ml (IQR 444, 566). There was a significant (*p* = 0.03) difference in median CD4 count for those who had progressed toCD4 counts of <350 cells/mm^3^ and those who had not. This difference indicated that CD4 count at entry was a confounder to be included in the logistic regression model during multivariate analyses. Daily income available for expenditure was found to be significantly (*p* = 0.04) associated with disease progression. There was no significant (*p* ≤ 0.05) association found at bivariate analyses of age, gender, level of education, housing and disease progression by CD4 count decline. As illustrated in Table 2.

**Table 2:** Demographic and socio-economic characteristics associated with HIV disease progression

Independent variable	Disease progression after two years *n* (%)	No Disease progression after two years *n* (%)	*p* value
Sex			
Male	21 (34.4)	40 (65.6)	0.058
Female	57 (22.7)	194 (77.3)	
Age			
24 years	12 (22.2)	42 (77.8)	0.685
25-34years	42 (24.0)	133 (76.0)	
35-44years	20 (30.8)	45 (69.2)	
>45 years	4 (22.2)	14 (77.8)	
Level of education			
None	4 (20)	16 (80)	0.754
Primary	30 (23.4)	98 (76.6)	
Secondary	28 (25.2)	83 (74.8)	
Tertiary	16 (30.2)	37 (69.8)	
Housing settlement			
Informal settlement	27 (23.3)	89 (76.7)	0.438
Formal settlement	51 (26.7)	140 (73.3)	
Not sure	0 (0)	5 (100)	
Daily income for expenditure			
Less than 1$	44 (32.6)	91 (67.4)	0.004
Between 1$-5$	30 (22.9)	101 (77.1)	
>5$	4 (8.7)	42 (91.3)	
Treatment arm			
Placebo	45 (31.7)	97 (68.3)	0.013
Acyclovir	33 (19.4)	137 (80.6)	

### Correlates of disease progression

At multivariate analysis, slower disease progression was associated significantly (*p* = 0.01) with reported available daily income for expenditure of more than US$5; [adjusted Odds Ratio (aOR) 4.6 (95 % Confidence Interval (CI) 1.4–14.4] as compared to incomes of < $1. However this association was not significant (*p* = 0.057) at available daily incomes of between $1–5. CD4 count levels at enrolment remained a significant (*p* < 0.001) predictor of disease progression (aOR 0.3 [95 % CI 0.2–0.4]. All the other variables in the model which included age, sex and clinical trial intervention were not independently associated with disease progression. Similarly, a significantly smaller proportion (19.4 %) of the participants who had received acyclovir as the clinical trial intervention had progressed compared to those in the placebo group (31.7 %), *p* = 0.013. As illustrated in Table 3.

**Table 3:** Predictors of HIV disease progression at multivariate analysis

Variable	Crude Odds Ratio (cOR)	95 % CI	*P* value	Adjusted Odds Ratio (aOR)	95 % CI	*p* value
Gender						
Female	0.6	0.3, 1.0	0.058	0.7	0.3, 1.5	0.354
Male	Ref			Ref		
Age in years	1.01	0.98, 1.04	0.600	0.99	0.95, 1.03	0.497
Expenditure/day						
< US$1	5.1	1.7, 15.1	0.003	4.5	1.4, 14.1	0.010
US$ 1-5	3.1	1.0, 9.4	0 .043	3.1	1.0, 9.7	0.057
>US$ 5	Ref			Ref		
Treatment group						
Acyclovir	0.5	0.3, 0.9	0.013	0.6	0.3, 1.0	0.052
Placebo	Ref			Ref		
CD4 at baseline/enrolment CD4 (cube root transformation)	0.3	0.2, 0.4	<0.0	0.3	0.2, 0.4	<0.001

## Discussion

In this study there were more HIV- infected individuals, who showed progression based on WHO clinical staging criteria compared to those showing progression based on CD4 count criteria. This is similar to studies in the PRE-HAART era [15] where disease progression was reported earlier when defined by laboratory criteria rather than by the development of an opportunistic infection.

This study found a strong association between daily incomes and disease progression portrayed by a decrease in CD4 counts to below 350 cells/mm^3^ (*p* = 0.026 CI 95 %). This progression occurred in spite of the optimal provision of free HIV care and treatment for opportunistic infections and prophylaxis using cotrimoxazole or dapsone. Income as a determinant of disease progression was strengthened by finding of an association between higher daily income available for expenditure and delayed disease progression. The acyclovir arm had been reported as having displayed delayed disease progression in the larger study [12]. Studies in America and Canada [5, 6] have reported a similar association between low incomes and more rapid HIV disease progression. Additional evidence shows, higher rates of disease progression after seroconversion among individuals with low incomes levels prior to infection [9]. The association between income and disease progression can therefore not be explained by a reduction of income due to HIV infection and morbidity. We postulate that micronutrient deficiencies which are common in low-income countries and have been proven to compromise the immune systems of HIV infected individuals [16] may play a role in the rate of disease progression observed [16]. Even with most treatment programmes providing multivitamin supplementation to individuals in care and treatment, People Living with HIV (PLWHIV) still bear a heavy burden of dietary micronutrient supplementation which is affected by the daily income available for expenditure. This contributes to the [16] weakening of the immune system and the depletion of CD4 cells resulting in faster disease progression. Micronutrient deficiency in the diet may explain the strong association found in this study between income available for daily expenditure and disease progression. Even with HAART provision, low income levels continue to be associated with poor health outcomes. In Kenya the inability to pay for transport to a HAART provision centre results in poor uptake of HAART [17]. Similarly, a study in British Columbia also reported the inability to pay for transport to a treatment centre as a cause of poor treatment outcomes among individuals with low-income [18]. The cost of transport to a treatment centre may therefore be another reason for the association between HIV disease progression and daily income available for expenditure.

Studies in Tanzania, Uganda and France [5, 19, 20] have found higher rates of disease progression in individuals above 40 years. This study did not find an association (*p* = 0.68; *p* < 0.05) between age, and disease progression. The finding of age as a non-determinant of disease progression in this study was most likely due to only 9 % of the participants’ being above 40 years. This study also found no association (*p* = 0.06; *p* < 0.05) between sex and disease progression measured using WHO criteria or a reduction in CD4 counts. This finding was corroborated by findings of various studies [6, 7] including a meta-analysis of 23 cohorts from Europe, Australia, and Canada which reported no association between sex and HIV disease progression [7]. The lack of association between level of education and disease progression in this study corresponds to findings in other similar studies of HAART naive individuals [5, 8]. Associations have been found in individuals already on HAART [21] with increased level of education. This delayed progression is attributed to the empowered attitude towards treatment and care resulting from higher levels of education. This did not apply to this study which focused on the period before initiation of HAART.

This study is unusual as it integrated widely accepted definitions of poverty [14] during analysis allowing comparison of results among other low and middle income countries within a global context. The study was conducted in a cohort of HIV positive individuals in serodiscordant unions who were all participating in a controlled trial. This may have introduced some level of selection bias though it is important to note that progression of disease is not expected to be different from other HIV positive individuals. The retrospective study design limited the socioeconomic indicators available to those captured during data collection for HSV2/HIV1 study clinical trial as this study was retrospective [12].

The data presented here however indicates that poverty, defined as the level of daily income available for expenditure, influences pre-HAART HIV disease progression and deserves consideration as a contextual factor in HIV disease progression. Additionally, impoverished populations may benefit from prioritization during implementation of WHO guidelines.

## Conclusions

The association reported between daily income available for daily expenditure and HIV disease progression indicates that economic empowerment should be considered as a possible contributor to better health outcomes in HIV infected individuals. We recommend prioritization of populations with the lowest daily available incomes for expenditure in low and middle income countries during the implementation of HAART scale up.
